# *Fzd4* Haploinsufficiency Delays Retinal Revascularization in the Mouse Model of Oxygen Induced Retinopathy

**DOI:** 10.1371/journal.pone.0158320

**Published:** 2016-08-04

**Authors:** Michael H. Ngo, Joanna Borowska-Fielding, Godfrey Heathcote, Sara Nejat, Melanie E. Kelly, Christopher R. McMaster, Johane M. Robitaille

**Affiliations:** 1 Department of Pharmacology, Dalhousie University, Halifax, NS, Canada; 2 Department of Pathology, Dalhousie University, Halifax, NS, Canada; 3 Department of Ophthalmology and Visual Sciences, Dalhousie University and the IWK Health Centre, Halifax, NS, Canada; 4 Department of Pathology and Molecular Medicine, McMaster University, Hamilton, ON, Canada; Monash University, Melbourne, Australia, AUSTRALIA

## Abstract

Mutations in genes that code for components of the Norrin-FZD4 ligand-receptor complex cause the inherited childhood blinding disorder familial exudative vitreoretinopathy (FEVR). Statistical evidence from studies of patients at risk for the acquired disease retinopathy of prematurity (ROP) suggest that rare polymorphisms in these same genes increase the risk of developing severe ROP, implying that decreased Norrin-FZD4 activity predisposes patients to more severe ROP. To test this hypothesis, we measured the development and recovery of retinopathy in wild type and *Fzd4* heterozygous mice in the absence or presence of ocular ischemic retinopathy (OIR) treatment. Avascular and total retinal vascular areas and patterning were determined, and vessel number and caliber were quantified. In room air, there was a small delay in retinal vascularization in *Fzd4* heterozygous mice that resolved as mice reached maturity suggestive of a slight defect in retinal vascular development. Subsequent to OIR treatment there was no difference between wild type and *Fzd4* heterozygous mice in the vaso-obliterated area following exposure to high oxygen. Importantly, after return of *Fzd4* heterozygous mice to room air subsequent to OIR treatment, there was a substantial delay in retinal revascularization of the avascular area surrounding the optic nerve, as well as delayed vascularization toward the periphery of the retina. Our study demonstrates that a small decrease in Norrin-Fzd4 dependent retinal vascular development lengthens the period during which complications from OIR could occur.

## Introduction

The retina is a thin layer of neural tissue lining the back of the eye responsible for sensing visual stimuli. During development, the retinal vasculature is initiated by endothelial sprouts that lay down the primary vessels that project radially from the optic disc to the retinal periphery to form the inner or primary vascular plexus. From this plexus, secondary vessels penetrate the retina to form the outer or tertiary plexus, thus laying down a vascular supply on either side of the central layer of neurons [1]. Patterning of the retinal vasculature is controlled by guidance cues driven initially by tissue hypoxia that is sensed by tip cells, specialized endothelial cells at the front end of the growing vasculature. Tip cells migrate along a pre-existing astrocyte network with endothelial stalk cells following the tip cells. Once in place, the primary vasculature matures into arteries and veins, the nascent network is pruned, and the blood-retina barrier is formed.

Retinopathy of prematurity (ROP) is a disorder that results in a defect in vascular development of the retinas of infants who are born prematurely. ROP is a leading cause of childhood blindness in developed countries and an emerging problem in developing nations. It is estimated that blindness affects at least ~20,000 prematurely born infants worldwide each year, of which at least 50% of cases are attributed to ROP [2, 3]. Infants with ROP have avascular zones of retina and are at risk of developing secondary neovascularization with subsequent retinal detachment. Treatments aiming to reduce the risk of vision loss in severe ROP include laser and anti-VEGF intraocular injection (*e*.*g*. bevacizumab). Recent studies suggest that, despite current treatments, vision of 20/40 or better (mild to no vision loss) occurs in only 1/3 of patients; most will be left with visual impairment while 1/4 will be legally blind [4]. The current visual outcomes in infants with severe ROP underscore the need to improve our understanding of the mechanisms that lead to blinding complications.

Several risk factors have been identified in association with severe ROP. It has been suggested that part of the course of ROP is influenced by genetic factors [5], with evidence of molecular links between ROP and genes that cause familial exudative vitreoretinopathy (FEVR) [6]. FEVR is an inherited developmental blinding disorder with an estimated incidence of 1:10,000. Similar to ROP, babies born with mutations in genes that cause FEVR present with hypovascularization of the retina due to the failure of peripheral retinal vascularization followed by secondary aberrant neovascularization [7–10], with severe forms of FEVR resulting in bilateral congenital retinal folds or retinal detachment [11–16]. FEVR is caused by mutations in six known genes, *FZD4* [17, 18], *TSPAN12* [19–21], *LRP5* [22], *NDP* [23–26], *ZNF408* [27] and *KIF11* [28, 29]. Four of the FEVR genes encode a ligand (norrin encoded by *NDP*), its receptor (encoded by *FZD4*), and two co-receptors (encoded by *LRP5* and *TSPAN12*). Rare polymorphisms in three of the known FEVR genes, *NDP*, *FZD4* and *LRP5* have been identified in patients with severe ROP in various populations [4, 30–38] suggesting that small defects in Norrin-FZD4 signaling may affect the development of ROP. Mouse models of FEVR have provided insight into the genotype-phenotype relationships that regulate retinal vascular development. The null mouse models of FEVR are accurate mimics of the human disease, demonstrating a failure of peripheral retina vascularization in addition to abnormal patterning and patency of the formed vessels [39–41].

To approximate ROP, rodents are subjected to ocular ischemic retinopathy (OIR) by placing them in either high oxygen, or high-low oxygen, environments for 5–14 days. The OIR protocol is meant to recapitulate the periods of relative hyperoxic and hypoxic oxygen phases following exposure to the extrauterine environment in prematurely born infants. We determined the extent of vascularization, vaso-obliteration and revascularization, in wild type and Fzd4 heterozygous (*Fzd4*^+/-^) mice in the absence and presence of exposure to an OIR protocol. *Fzd4*^+/-^ mice had a slight delay in ocular vascular development in room air. Importantly, we determined that *Fzd4*^+/-^ mice subjected to OIR displayed a substantive defect in revascularization following re-exposure to room air compared to wild type mice. This is the first functional demonstration that a small decrease in Norrin-Fzd4 dependent retinal vascular development lengthens the period of vascular recovery subsequent to OIR. Our functional results in mice align with the previous genetic studies in patients that observed a greater prevalence of rare polymorphisms in the genes encoding components of the Norrin-FZD4 pathway in infants that developed more severe ROP.

## Materials and Methods

### Ethics Statements on Mouse Models and Animal Care

Experiments were carried out using pups bred from *Fzd4*^+/-^ male and female breeders (B6;129-*Fzd4*^*tm1Nat*^/J; The Jackson Laboratory, Bar Harbor, ME, USA). Both male and female mice were used in this study. All procedures were performed in accordance with the Canadian Council on Animal Care and approved by the Research Ethics Board for Laboratory Animal Research at Dalhousie University. For OIR studies, mice were exposed to 65% O_2_ for the first three days followed by room air for 24 hrs and 65% O_2_ for the final 24 hrs. No mice became ill or died prior to the experimental endpoint. Animals were anesthetized with isoflurane prior to euthanization and retina isolation. For each condition, a minimum of three litters per group (range: 3–5 litters) was compared yielding at least 6 eyes per group (range: 6–11 eyes). Weights for all mice were measured and compared at postnatal day 12 (P12), P17 and P25.

### Mouse Genotyping

DNA was extracted (AccuStart^TM^ II Mouse Genotyping Kit, Quanta Biosciences) from ear tissue. Mouse *Fzd4* gene primers were obtained from Integrated DNA Technologies, Inc. Primers for the wild type *Fzd4* gene were WT F 5’-TGG AAA GCG TAA TGG TCA AGA TCGG and WT R 5’-AGA ATT CAC CAA TCG GTT AGA ACAC. Primers for the *Fzd4* gene knockout were Mut F 5’-TGT CTG CTA GAT CAG CCT CTG CCG and Mut R 5’-CAT CAA CAT TAA ATG TGA GCG AGT. Mouse genotype was determined by PCR from genomic DNA.

### Retina Isolation

Retinas were isolated and fixed in Dulbecco's phosphate buffered saline containing 0.1g/l MgCl_2_ and 0.133g/l CaCl_2_ (Sigma-Aldrich) in 4% paraformaldehyde (Cedarlane) for 2 hours at room temperature. Fixation was quenched with phosphate buffered saline containing 0.3% Triton X-100 and 10% goat serum (Sigma-Aldrich) for 1 hour at room temperature. To visualize the vascular endothelium, retinas were stained with Alexa Fluor 594-conjugated GS-IB4 lectin (20μg/ml; Molecular Probes) at 4°C overnight. Retinas were washed three times with phosphate buffered saline containing 0.1% Triton X-100 and flat-mounted (Fluoromont-G, Sigma-Aldrich).

### Retinal Vasculature Imaging and Analysis

Retinas were visualized using a Zeiss Axio Imager Z2 microscope containing a digital stage and the MosaiX software program in Axiovision 4.8. All analyses were performed on images captured at 10x magnification with the same image acquisition settings. Avascular and total areas were quantified in CS Photoshop 6 to give a percentage of vaso-obliteration. Vascularization was quantified as a percentage of the total area using Image J software program and NV_Swift plugin [42]. For vessel count, whole mount retinas were analyzed using ImageJ software program. For each quadrant, three line segments were placed at specified locations: adjacent to the optic nerve (proximal), adjacent to the periphery of the retina (distal), and midway between the proximal and distal lines (mid). The number of vessels crossing each line in four different quadrants was counted and averaged at each of the three locations. The width of primary vessels adjacent to the optic disc was calculated using Image J. One eye per animal (the right eye) was used for image analysis. To visualize whether vessels were protruding into multiple layers, whole mount retinas were imaged using Zeiss LSM 510 Meta Laser Scanning Confocal microscope at 25x. Slices were 1 micron apart. Z-stacks were analyzed using ImageJ Software program.

### Statistical Analyses

Imaging analyses are expressed as mean ± standard error of the mean (SEM) and *P* values were calculated using a one-way ANOVA unpaired t-test. The animal weights were described as mean ± standard error (SE) and compared using the *t*-test with a two-tailed distribution. Results were considered significant if *P* < 0.05.

## Results

### Retina Vascular Development in Wild Type and *Fzd4*^+/-^ Mice

We determined the status of retinal vascular development in *Fzd4*^+/-^ mice raised in room air to determine if *Fzd4* haploinsufficiency had an effect on vascular development. Comparisons were made with wild type littermates including the average number and caliber of vessels by determining the number of vessels per mm and the percentage of vessels >50 μm (a 50 μm cutoff was chosen to represent the width of major primary vessels (*i*.*e*. main arterioles from the inner plexus) based on observation of normal adult retina vessels. Vessel number and caliber were analyzed at three locations (proximal, middle, and distal) based on distance from the optic nerve. Mice were analyzed at P12, P17 and P25, these time points were chosen as they are typically used to evaluate retinal pathology observed in the mouse OIR model.

**Fig 1:**
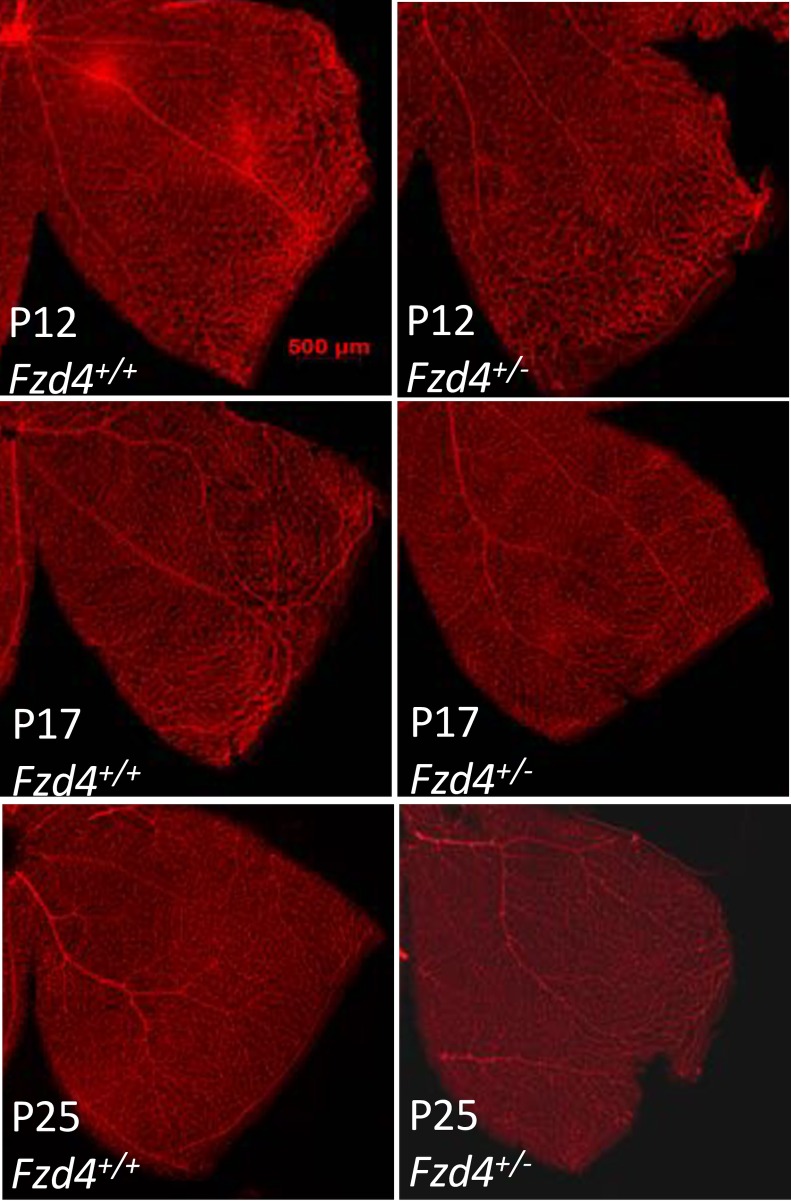
Analysis of retinal vasculature during development for wild type and *Fzd4*^+/-^ mice under normoxic conditions. **(A)** Wild type and **(B)** *Fzd4*^+/-^ mice were raised in room air. At P12, P17, and P25 whole mounted retinas were stained with Alexa Fluor 594-conjugated GS-IB4 lectin and visualized using confocal microscopy. The yellow square in the image on the left is enlarged in the panel on the right.

**Fig 2:**
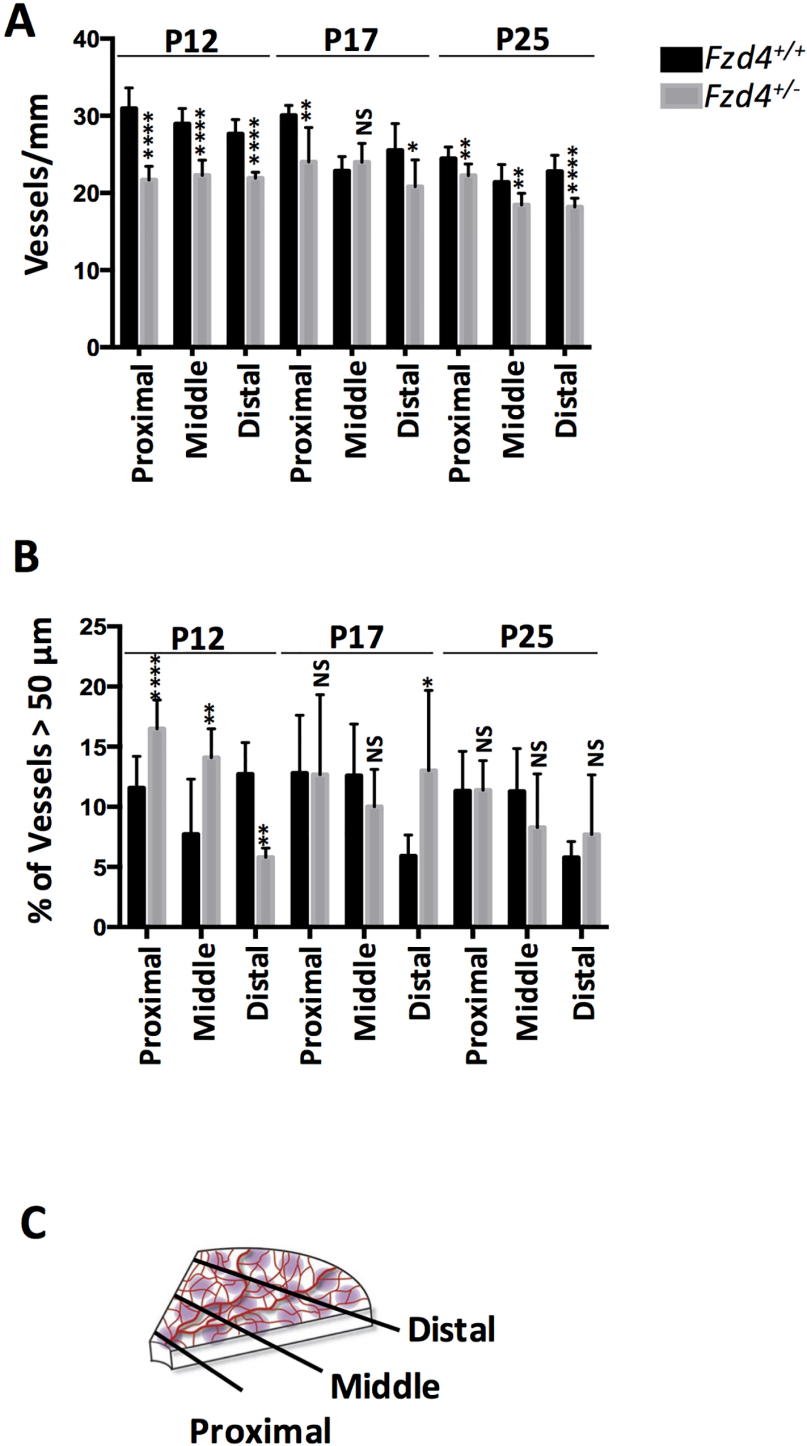
Changes in vessel number and calibre under normoxic conditions for wild type and *Fzd4*^+/-^ mice. Mice were raised in room air and at P12, P17, and P25 whole mounted retinas were stained with Alexa Fluor 594-conjugated GS-IB4 lectin and visualized using confocal microscopy. (**A**) For vessel count, profile plots were created by drawing three lines in each of the four quadrants at three specified locations: adjacent to the optic nerve (proximal), adjacent to the periphery of the retina (distal) and midway between the proximal and distal lines (middle). The number of vessels crossing each line in each quadrant was counted. (**B**) The width of vessels at proximal, mid, and distal regions from the optic nerve was calculated using Image J. A 50 μm cutoff was chosen to represent the width of major primary vessels and calculated as a percentage of total vasculature. (**C**) A schematic representation of vessel number and caliber determination. Line segments were drawn perpendicular to retinal quadrants at the proximal, middle and distal regions. Imaging analyses are expressed as mean ± standard error of the mean (SEM) and *P* values were calculated using a one-way ANOVA unpaired t-test. *P<0.05, **<0.01, ***<0.001, ****<0.0001. NS, not significant.

At P12, wild type mice had more vessels than *Fzd4*^+/-^ mice with 31 vessels per mm versus 22 in the proximal, 28 versus 20 in the mid periphery, and 28 versus 22 in the distal retinal regions, respectively (Figs 1 and 2). Retinal vessel caliber measured as a percentage of vessels >50 μm was increased in the proximal and mid regions in *Fzd4*^+/-^ mice compared to wild type mice at P12, while vessel caliber was decreased in the distal region (Fig 2). At P17 and P25, the *Fzd4*^+/-^ mice were beginning to normalize vessel number and caliber, with vessel caliber fully normalized by P25 and number of vessels approaching normalization. Specifically, in wild type versus *Fzd4*^+/-^ mice, at P17 there were 30 vessels per mm versus 25 in the proximal, 24 versus 25 in the mid periphery, and 25 versus 21 in the distal region, while at P25 there were 24 vessels per mm versus 23 in the proximal, 22 versus 18 in the mid, and 23 versus 19 in the distal region of the retina (Figs 1 and 2). For vessel caliber, at P17 there was no change in proximal or mid peripheral regions while there was an increase from 6% to 15% in vessels >50 μm in the *Fzd4*^+/-^ compared to wild type mice. By P25 vessel caliber changes had been resolved in all regions of the retina while there was still a small but statistically significant decrease in the number of vessels/mm across all regions of the retina in the *Fzd4*^+/-^ mice (Figs 1 and 2).

**Fig 3:**
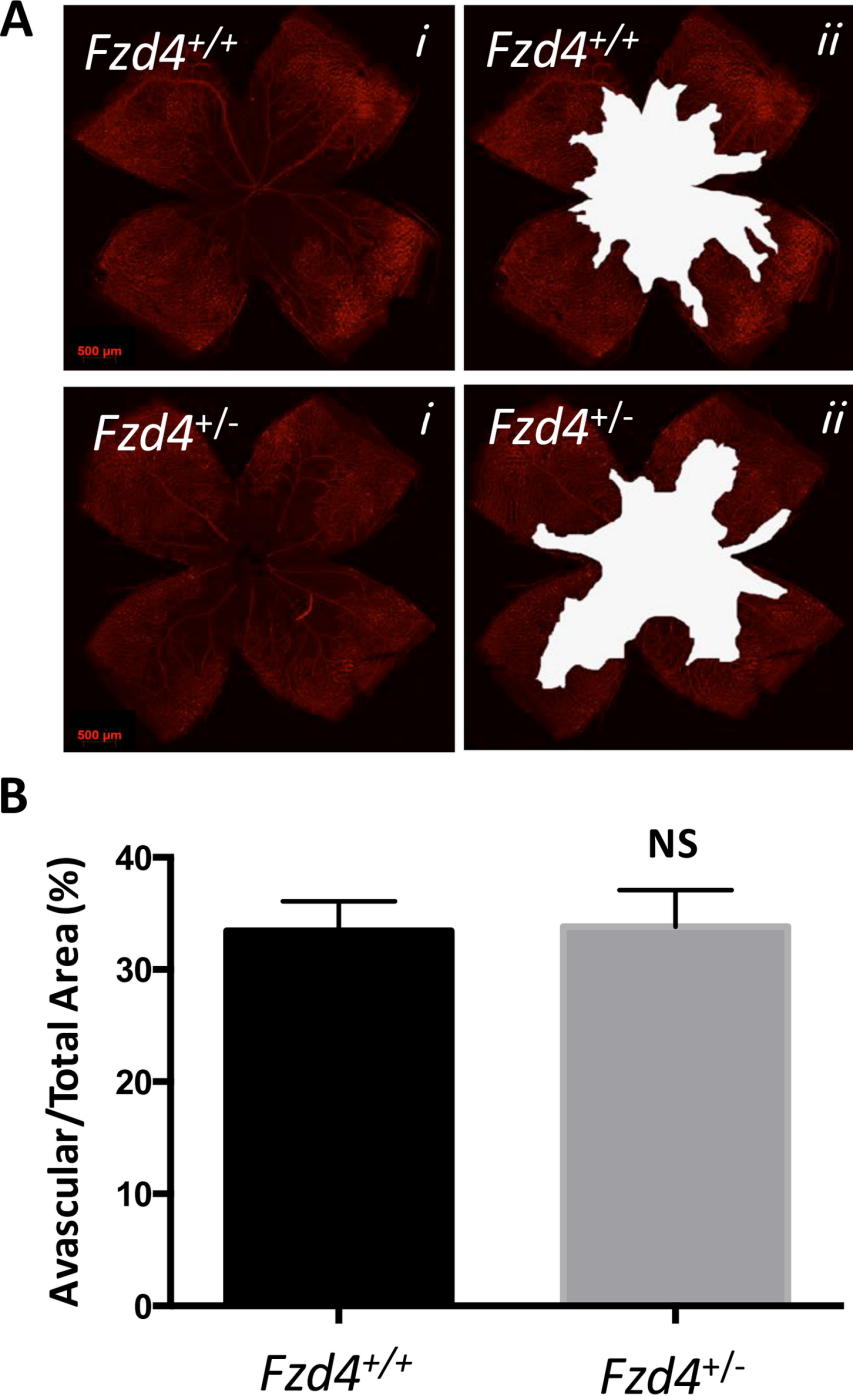
Retinal vasculature development into the deep and intermediate layers of wild type and *Fzd4*^+/-^ mice under normoxic conditions. Whole mount retinas of *Fzd4*^+/+^ and *Fzd4*^+/-^ under normoxic conditions were stained with isolectin-594 and imaged at 25x magnification using a Zeiss LSM 510 Meta laser scanning confocal microscope. Z-stacks representing vascular growth in the deep and intermediate layers at the mid periphery were generated using 1 micron slices. Z-stacks were analyzed using the ImageJ software program. Green bars represent the X-axis, blue bars represent the Y-axis and purple represent the Z-axis. White arrows indicate examples of vessel branching in retinal layers.

Retinal vascular development proceeds three dimensionally into intermediate and deep layers. To determine whether *Fzd4*^+/-^ mice had a delay in vasculature development into their intermediate and deep layers, whole mount retinas of mice were analyzed by taking Z-stack images of the retina flat mounts at P12, P17, and P25 (Fig 3). Z-stacks were compiled to produce XY, XZ and YZ dimensions. White arrows indicate vessel growth within vascular layers. There was no observable difference between *Fzd4*^+/+^ and *Fzd4*^+/-^ mice at P12, P17 and P25 under normoxic conditions as vessel branching moved through the intermediate layer and into the the deep layer of the retina. For both *Fzd4*^+/+^ and *Fzd4*^+/-^ mice at P12 and P17, vessels were detected in the inner and middle layer of the retina with deeper vessels emerging by P25.

### Effect of Exposure to O_2_ in the Mouse OIR Model

The standard mouse OIR model used to mimic ROP exposes mice to 75% O_2_ for five days from P7 to P12; this causes vaso-obliteration of the central retina surrounding the optic nerve. At P12, mice are re-exposed to room air resulting in neovascularization at the vaso-obliterated edges (maximum effect at P17), revascularization of the vaso-obliterated area, as well as continued vascularization toward the distal region of the retina [43]. This level of O_2_ is used to guarantee all mice in the cohort develop ROP-like vaso-obliteration. The standard mouse protocol did not demonstrate a difference in the effects of OIR between *Fzd4*^+/+^ and *Fzd4*^+/-^ mice. Given the small but significant effects of haploinsufficiency in *Fzd4*^+/-^ mice under normoxic conditions, and to allow for comparison of the wild type and *Fzd4*^+/-^ mice to human genetic data that suggested a small decrease in Norrin-FZD4 mediated vascular development could increase risk for severe ROP, we wanted to establish the minimum oxygen exposure (the critical tipping point) that elicited vaso-obliteration in mice. At 65% O_2_ we observed the same level of vaso-obliteration as was previously seen in wild type mice exposed to 75% O_2_ [44, 45] with approximately one third of the retina ablated. Conversely, no retinopathy was detected when mice were exposed to less than 60% O_2_. We found that providing an additional insult by introducing one day of exposure to room air at P10, followed by 65% O_2_ for one day at P11, prior to returning mice to room air at P12 produced a reliable and consistent vaso-obliteration phenotype (Fig 3). Varying high and low O_2_ levels is similar to what is done in the rat model of OIR.

### A Delay in Retinal Vascular Recovery in *Fzd4*^+/-^ Mice Subjected to OIR

**Fig 4:**
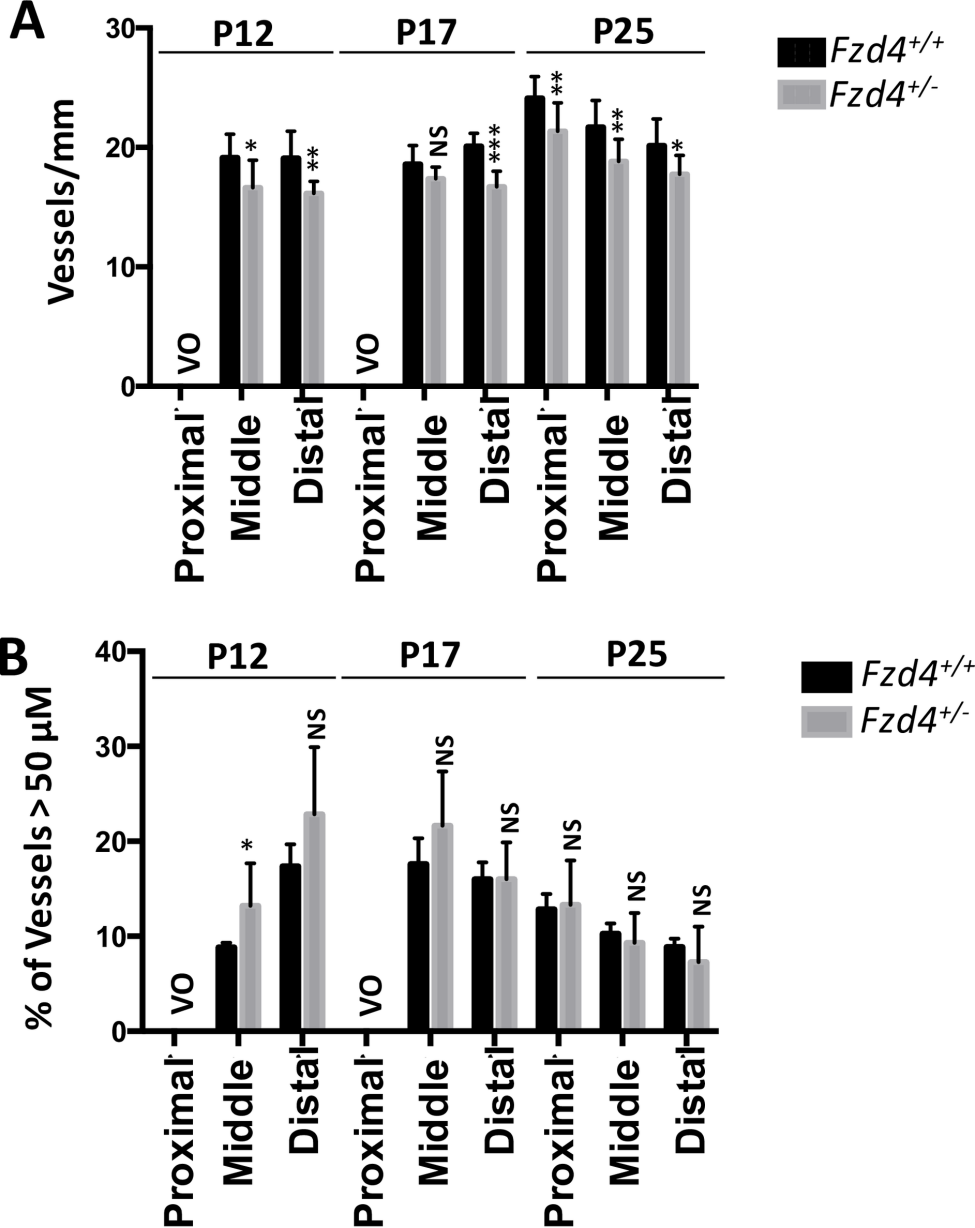
Retinal vaso-obliteration of wild type and *Fzd4*^+/-^ mice after ocular ischemic retinopathy. Wild type and *Fzd4*^+/-^ mice were exposed to our OIR model from P7 to P12. (**A**) At P12 whole mounted retinas were stained with Alexa Fluor 594-conjugated GS-IB4 lectin and visualized using confocal microscopy (panel *i*). Vaso-obliterated areas are shown in white (panel *ii*). (**B**). Quantification of the vaso-obliterated area. Imaging analyses are expressed as mean ± standard error of the mean (SEM) and *P* values were calculated using a one-way ANOVA unpaired t-test. NS, not significant.

**Fig 5:**
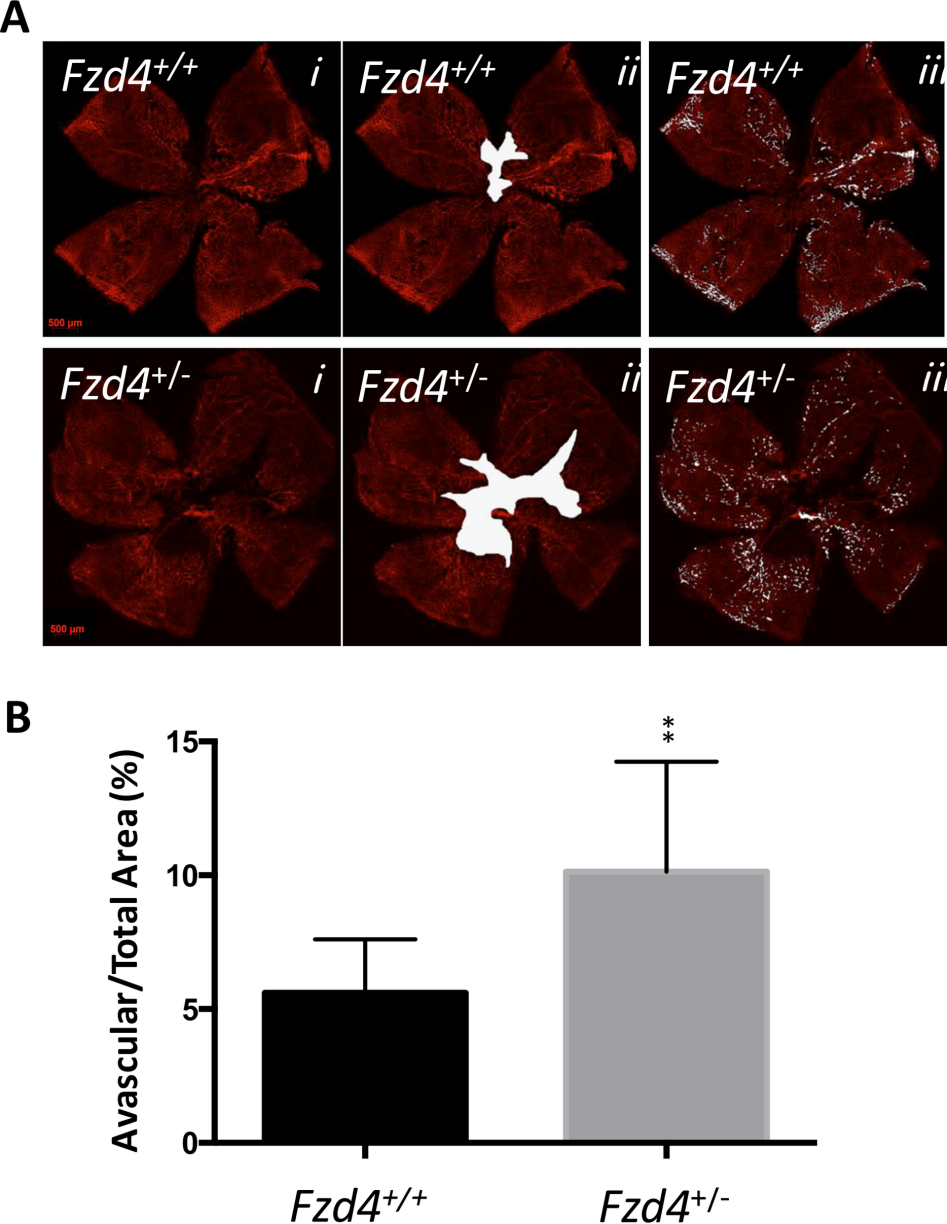
Decreased vessel number and increased vessel caliber in *Fzd4*^+/-^ mice after ocular ischemic retinopathy. Comparison of vessel number and caliber for wild-type and *Fzd4*^+/-^ mice after ocular ischemic retinopathy at P12, P17 and P25. (**A**) For vessel count, the number of vessels at lines in each quadrant at proximal, mid, and distal regions from the optic nerve was determined. (**B**) The width of vessels at proximal, mid, and distal regions from the optic nerve was determined was calculated using Image J. A 50 μm cutoff was chosen to represent the width of major primary vessels. Imaging analyses are expressed as mean ± standard error of the mean (SEM) and *P* values were calculated using a one-way ANOVA unpaired t-test. * P<0.05, **<0.01, ***<0.001. VO, vaso-obliterated. NS, not significant.

The effect of our modified OIR protocol on the area of vaso-obliteration, revascularization of the vaso-obliterated area, continued vascularization toward the periphery, and number and caliber of vessels was assessed at P12, P17 and P25 in wild type and *Fzd4*^+/-^ mice to determine if the defects observed in vascular development in *Fzd4*^+/-^ mice would be exacerbated. When mice were removed from the high O_2_ environment at P12, there was no difference in the area of vaso-obliteration between *Fzd4*^+/-^ and wild type mice with both having 34% of the retina vaso-obliterated (Fig 4). There was a modest decrease in the number of vessels in *Fzd4*^+/-^ mice in the mid peripheral and distal regions of the retina compared to wild type (Fig 5). Also, vessel caliber was increased in *Fzd4*^+/-^ mice compared to wild type, from 9% to 13% in the mid periphery and 18% to 25% in the distal region (Fig 5).

**Fig 6:**
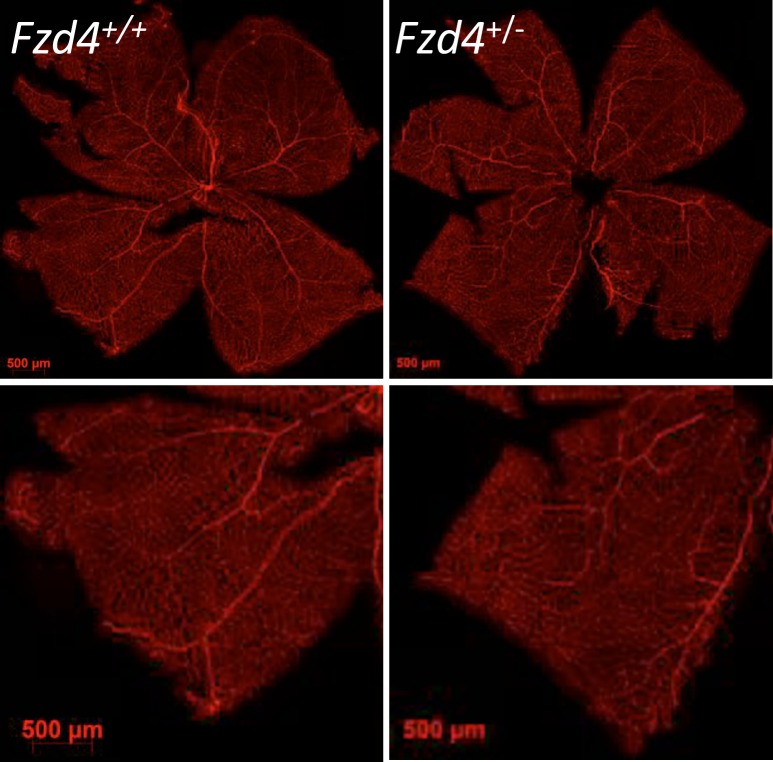
Delayed revascularization in *Fzd4*^+/-^ mice after ocular ischemic retinopathy. Wild type and *Fzd4*^+/-^ mice were exposed to our OIR model from P7 to P12 and returned to room air. At P17 (**A**) whole mounted retinas were stained with Alexa Fluor 594-conjugated GS-IB4 lectin and visualized using confocal microscopy (panel *i*), vaso-obliterated areas are shown in white (panel *ii*). (**B**). Quantification of the vaso-obliterated area. Imaging analyses are expressed as mean ± standard error of the mean (SEM) and *P* values were calculated using a one-way ANOVA unpaired t-test. **P<0.01.

**Fig 7:**
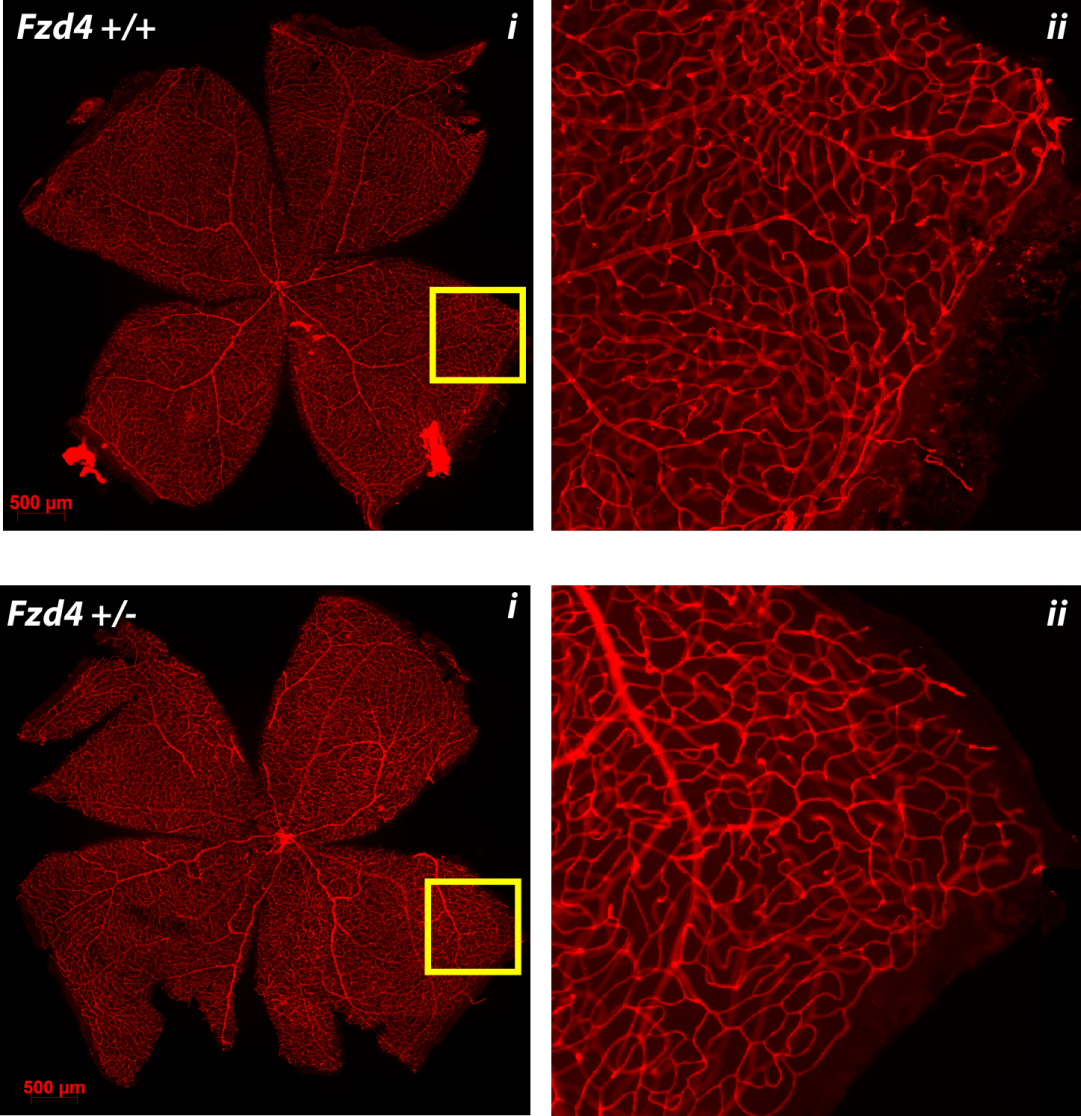
Toward normalization of vascularization of *Fzd4*^+/-^ mice after ocular ischemic retinopathy. Wild type and *Fzd4*^+/-^ mice were exposed to our OIR model from P7 to P12 and returned to room air. At P25 whole mounted retinas were stained with Alexa Fluor 594-conjugated GS-IB4 lectin and visualized using confocal microscopy. The yellow square in the image on the left is enlarged in the panel on the right.

The most obvious and important difference between wild type and *Fzd4*^+/-^ mice exposed to the OIR model was observed at P17, during the recovery phase. Wild type mice had 6% of residual vaso-obliterated retina while *Fzd4*^+/-^ mice had 10%, a 66% increase compared to wild type (Fig 6). This indicates that, compared to *Fzd4*^+/+^mice, *Fzd4*^*+/-*^ mice had a substantially decreased ability to revascularize ablated retina. It was also clear that new vessel growth had progressed to the distal periphery in wild type mice at P17 and this was delayed in the *Fzd4*^*+/-*^ mice (Figs 5 and 6A), as determined by number of vessels/mm and the intensity of Alexa Fluor 594-conjugated GS-IB4 lectin staining. There was very little neovascularization in either wild type or *Fzd4*^*+/-*^ mice exposed to our modified OIR protocol. At P25, the small decrease in vessel number measured in *Fzd4*^+/-^ mice was retained across all areas of the retina while vessel caliber was similar to wild type (Figs 5 and 7), consistent with delayed revascularization in both the proximal and distal regions of the retina in *Fzd4*^*+/-*^ mice exposed to OIR.

**Fig 8:**
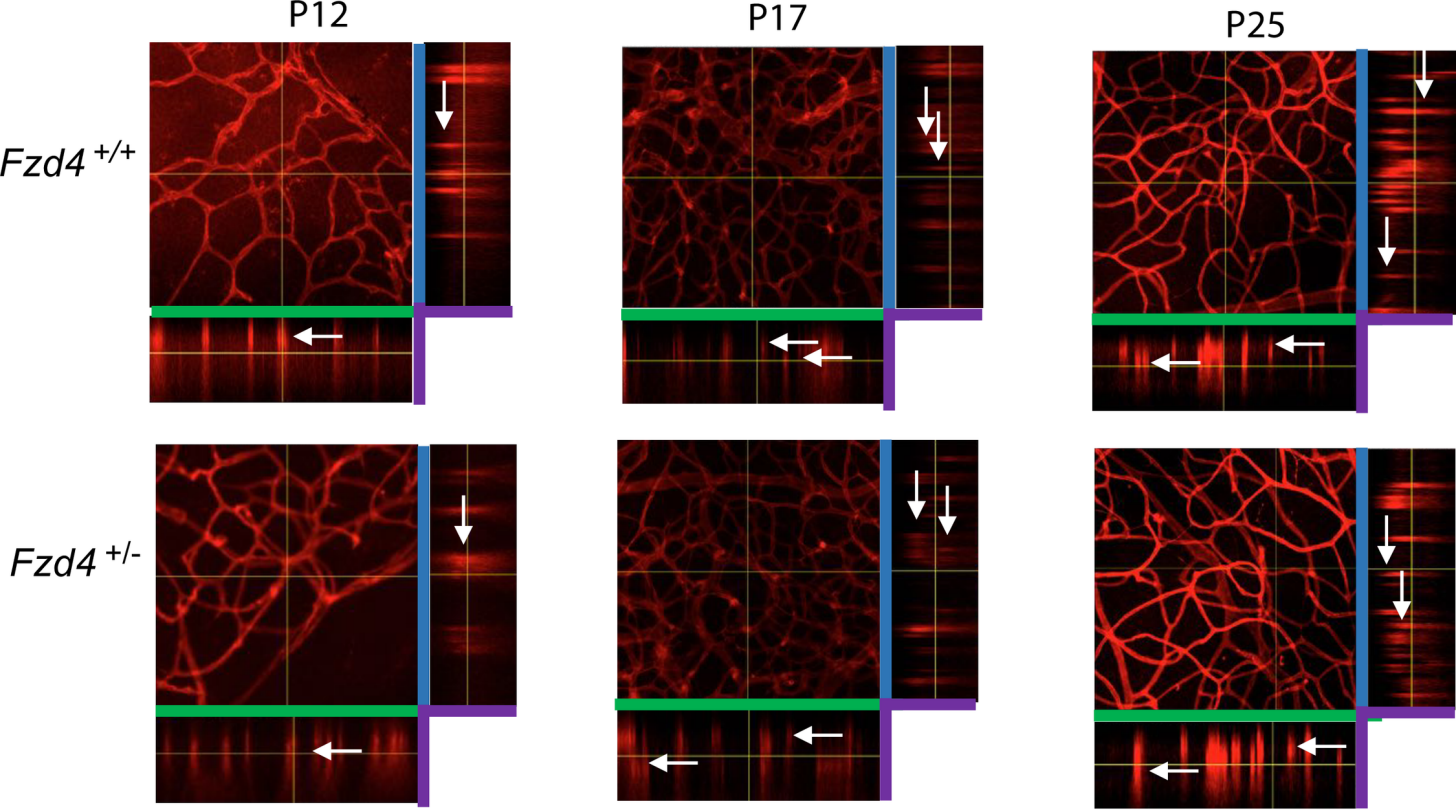
Retinal vasculature development into the deep and intermediate layers of wild type and *Fzd4*^+/-^ mice after ocular ischemic retinopathy. Whole mount retinas of *Fzd4*^+/+^ and *Fzd4*^+/-^ subjected to the ocular ischemic retinopathy protocol were stained with isolectin-594 and imaged at 25x magnification using a Zeiss LSM 510 Meta laser scanning confocal microscope. Z-stacks representing vascular growth in the deep and intermediate layers at the mid periphery were generated using 1 micron slices. Z-stacks were analyzed using the ImageJ software program. Green bars represent the X-axis, blue bars represent the Y-axis and purple represent the Z-axis. White arrows indicate examples of vessel branching in retinal layers.

We also determined whether retinal vessel growth in the intermediate and deep layers was altered in *Fzd4*^+/+^ and *Fzd4*^+/-^ mice under OIR conditions (Fig 8). *Fzd4*^+/+^ and *Fzd4*^+/-^ mice were compared to mice raised under normoxic conditions (Fig 3), and to each other, following OIR treatment. At P12, both the *Fzd4*^+/+^ and *Fzd4*^+/-^ mice displayed a delay in intermediate and deep layer development after OIR treatment compared to retinas from normoxic mice. At P17 and especially at P25, the *Fzd4*^+/+^ and *Fzd4*^+/-^ mice did form intermediate and deep layers, however, most vessels were within a single plane compared mice reared under normoxic conditions, suggesting a delay in the formation of these deeper layers resulting from prior exposure to OIR conditions.

As weight has been known to affect vaso-obliteration and vascular recovery in mice [46, 47] we compared weight of wild type and *Fzd4*^+/-^ mice at P12, P17, and P25 under normoxic and the modified OIR conditions. We did not observe differences in weight between the wild type and *Fzd4*^+/-^ mice (S1 Table).

## Discussion

In this study we sought to determine if haploinsufficiency of *Fzd4* affects ocular vascular development in the mouse, and if *Fzd4* haploinsufficiency alters the course of vascular changes observed during OIR. *Fzd4*^+/-^ mice had a small delay in vascular development in room air that resolved as the mice matured. Specifically, at P12 there was a decrease in vessel number in all areas of the retina and increased caliber in the proximal and mid peripheral areas in *Fzd4*^+/-^ mice. By P17 vessel number resolved slightly in *Fzd4*^+/-^ mice while there remained increased distal vessel caliber measured as a percentage of vessels > 50 μm. This is possibly due to a compensatory response to hypoxic conditions in the peripheral retina, or a relative delay in development of the microvascular bed (as opposed to the larger primary vessels in the inner plexus) resulting from delayed peripheral vascularization due to *Fzd4* haploinsufficiency. By P25, retinal vessel patterning and caliber was similar between wild type and *Fzd4*^+/-^ mice. *Fzd4*^+/-^ mice did not exhibit a delay in the formation of the deeper vascular layers at P12, P17 or P25 suggesting that *Fzd4* haploinsufficiency does not influence this aspect of vascular development under normoxic conditions.

We went on to use an OIR model to determine if the small defect we observed in the Norrin-Fzd4 mediated retinal vascular development in the *Fzd4*^+/-^ mice affects vaso-obliteration and subsequent revascularization. A main driver of our mouse OIR study was the previous observations that rare polymorphisms in the known FEVR genes *NDP*, *FZD4* and *LRP5* were enriched in patients with severe ROP [4, 30–38, 48]. The human data implied that a small decrease in Norrin-FZD4 signaling could alter the course of ROP. We determined that the vaso-obliterated area was unaffected in *Fzd4*^+/-^ mice compared to wild type mice, however, following re-exposure to room air *Fzd4*^+/-^ mice displayed a substantive delay in revascularization toward the proximal region of the retina, as well as a delay in vascularization toward to peripheral retina. Exposure of *Fzd4*^+/-^ mice to the modified OIR model does not increase neovascular development nor does it affect the emergence of the deeper retina layers compared to wild type mice. ROP in premature infants is due to disruption of normal vascularization of the developing retina, a process that normally requires full gestation for completion in humans. The two-phase hypothesis for the development of ROP consists of an initial period of relative hyperoxia (phase 1) with associated delays in vascularization, followed by retinal hypoxia due to absence of vessels in the developing retina (phase 2). This leads to aberrant neovascularization at the developing ROP edge putting the infant at risk of developing tractional retinal detachment. The lengthening of the period of vascular recovery we observed in *Fzd4*^+/-^ mice would allow for a longer time frame for which infants would be at risk of developing neovascularization. This is consistent with the observed rare polymorphisms identified in Norrin-FZD4 genes in premature infants with severe ROP, and suggests that in these infants a small decrease in Norrin-FZD4 mediated vascular development results in an increased risk for the development of blinding complications of ROP.

In support of our results are two studies where Norrin dose was increased in mouse models of OIR to drive Norrin-Fzd4 signaling. Ectopic overexpression of Norrin in transgenic mice (βB1-crystallin-norrin mice) exposed to the OIR protocol promoted vascular regrowth at P14 and P16 in a dose dependent fashion [49]. It also reduced vaso-obliteration suggesting increased norrin-Fzd4 signaling may offer protection against hyperoxic damage. More recently, Tokunaga et al [50] were able to rescue OIR in mice following a single intravitreal injection of Norrin at P14. They observed a significant decrease in the avascular area subsequent to the OIR protocol, and to a lesser degree a decrease in neovascularization at P17. They noted a progression of vascular recovery between P15 and P20 suggesting faster remodeling in Norrin treated animals.

Characterizing the effects of stressors on developmental angiogenesis improves our understanding of retinal vascular disease processes and can help identify potential therapeutic targets. ROP is a blinding disorder that affects prematurely born infants and is a leading cause of blindness, leaving an estimated 20,000 infants blind or severely visually impaired worldwide annually.[2] Current treatments for severe ROP including laser and anti-VEGF agents are helpful in decreasing the risk of blindness. Nevertheless, vision loss [51, 52], reduced visual fields [53, 54], persistent vascularization defects with risks for late complications [55–60] and concerns for potential systemic side effects [61–65] remain significant problems, highlighting the need to develop better predictors for infants at risk for more severe ROP to aid in prioritizing treatment to reduce lifelong visual impairment. Our studies suggest that screening for rare polymorphisms in FEVR genes could be a worthwhile endeavour to determine their predictive value for the development of more severe ROP. Treatments that aim to alter Norrin-Fzd4 signaling may decrease the risk of blindness in prematurely born infants by reducing the vulnerable time period during which blinding complications can occur.

## Supporting Information

S1 Table Weights of mice used in this study. (PDF)

## References

[1] Ye X, Wang Y, Nathans J. The Norrin/Frizzled4 signaling pathway in retinal vascular development and disease. Trends in molecular medicine. 2010;16(9):417–25. Epub 2010/08/07. 10.1016/j.molmed.2010.07.003 20688566 PMC2963063

[2] Blencowe H, Lawn JE, Vazquez T, Fielder A, Gilbert C. Preterm-associated visual impairment and estimates of retinopathy of prematurity at regional and global levels for 2010. Pediatric research. 2013;74 Suppl 1:35–49. Epub 2013/12/25. 10.1038/pr.2013.205 24366462 PMC3873709

[3] Wallace DK, Bremer DL, Good WV, Fellows R, Summers CG, Tung B, et al Correlation of recognition visual acuity with posterior retinal structure in advanced retinopathy of prematurity. Archives of ophthalmology. 2012;130(12):1512–6. Epub 2012/12/12. 10.1001/archopht.130.12.1510 . 22892757

[4] Haider MZ, Devarajan LV, Al-Essa M, Srivastva BS, Kumar H, Azad R, et al Retinopathy of prematurity: mutations in the Norrie disease gene and the risk of progression to advanced stages. Pediatr Int. 2001;43(2):120–3. 11285060 10.1046/j.1442-200x.2001.01361.x

[5] Flynn JT. The premature retina: a model for the in vivo study of molecular genetics? Eye. 1992;6 (Pt 2):161–5. . 1624038 10.1038/eye.1992.32

[6] Shastry BS. Genetic susceptibility to advanced retinopathy of prematurity (ROP). Journal of biomedical science. 2010;17:69 Epub 2010/08/27. 10.1186/1423-0127-17-69 20738858 PMC2933676

[7] van Nouhuys CE. Dominant exudative vitreoretinopathy and other vascular developmental disorders of the peripheral retina. Doc Ophthalmol. 1982;54(1–4):1–414. . 6897033 10.1007/BF00183127

[8] Ober RR, Bird AC, Hamilton AM, Sehmi K. Autosomal dominant exudative vitreoretinopathy. Br J Ophthalmol. 1980;64(2):112–20. . 7362811 10.1136/bjo.64.2.112 PMC1039360

[9] Canny CL, Oliver GL. Fluorescein angiographic findings in familial exudative vitreoretinopathy. Arch Ophthalmol. 1976;94(7):1114–20. . 947162 10.1001/archopht.1976.03910040034006

[10] Gow J, Oliver GL. Familial exudative vitreoretinopathy. An expanded view. Arch Ophthalmol. 1971;86(2):150–5. . 5571414 10.1001/archopht.1971.01000010152007

[11] Chang-Godinich A, Paysse EA, Coats DK, Holz ER. Familial exudative vitreoretinopathy mimicking persistent hyperplastic primary vitreous. Am J Ophthalmol. 1999;127(4):469–71. . 10218708 10.1016/s0002-9394(99)00003-3

[12] Nishimura M, Yamana T, Sugino M, Kohno T, Yamana Y, Minei M, et al Falciform retinal fold as sign of familial exudative vitreoretinopathy. Jpn J Ophthalmol. 1983;27(1):40–53. . 6855020

[13] van Nouhuys CE. Juvenile retinal detachment as a complication of familial exudative vitreoretinopathy. Fortschr Ophthalmol. 1989;86(3):221–3. . 2759534

[14] Shubert A, Tasman W. Familial exudative vitreoretinopathy: surgical intervention and visual acuity outcomes. Graefes Arch Clin Exp Ophthalmol. 1997;235(8):490–3. . 9285217 10.1007/BF00947005

[15] Qin M, Hayashi H, Oshima K, Tahira T, Hayashi K, Kondo H. Complexity of the genotype-phenotype correlation in familial exudative vitreoretinopathy with mutations in the LRP5 and/or FZD4 genes. Hum Mutat. 2005;26(2):104–12. . 15981244 10.1002/humu.20191

[16] van Nouhuys CE. Signs, complications, and platelet aggregation in familial exudative vitreoretinopathy. Am J Ophthalmol. 1991;111(1):34–41. . 1985487 10.1016/s0002-9394(14)76893-x

[17] Robitaille J, MacDonald ML, Kaykas A, Sheldahl LC, Zeisler J, Dube MP, et al Mutant frizzled-4 disrupts retinal angiogenesis in familial exudative vitreoretinopathy. Nat Genet 2002;32(2):326–30. 12172548 10.1038/ng957

[18] Robitaille JM, Zheng B, Wallace K, Beis MJ, Tatlidil C, Yang J, et al The role of Frizzled-4 mutations in familial exudative vitreoretinopathy and Coats disease. Br J Ophthalmol. 2011;95(4):574–9. 10.1136/bjo.2010.190116 21097938

[19] Junge HJ, Yang S, Burton JB, Paes K, Shu X, French DM, et al TSPAN12 regulates retinal vascular development by promoting Norrin- but not Wnt-induced FZD4/beta-catenin signaling. Cell. 2009;139(2):299–311. Epub 2009/10/20. 10.1016/j.cell.2009.07.048 . 19837033

[20] Poulter JA, Ali M, Gilmour DF, Rice A, Kondo H, Hayashi K, et al Mutations in TSPAN12 cause autosomal-dominant familial exudative vitreoretinopathy. American journal of human genetics. 2010;86(2):248–53. Epub 2010/02/18. 10.1016/j.ajhg.2010.01.012 20159112 PMC2820188

[21] Nikopoulos K, Gilissen C, Hoischen A, van Nouhuys CE, Boonstra FN, Blokland EA, et al Next-generation sequencing of a 40 Mb linkage interval reveals TSPAN12 mutations in patients with familial exudative vitreoretinopathy. American journal of human genetics. 2010;86(2):240–7. Epub 2010/02/18. 10.1016/j.ajhg.2009.12.016 20159111 PMC2820179

[22] Jiao X, Ventruto V, Trese MT, Shastry BS, Hejtmancik JF. Autosomal recessive familial exudative vitreoretinopathy is associated with mutations in LRP5. Am J Hum Genet. 2004;75(5):878–84. . 15346351 10.1086/425080 PMC1182117

[23] Berger W, van de Pol D, Warburg M, Gal A, Bleeker-Wagemakers L, de Silva H, et al Mutations in the candidate gene for Norrie disease. Hum Mol Genet. 1992;1(7):461–5. . 1307245 10.1093/hmg/1.7.461

[24] Chen ZY, Hendriks RW, Jobling MA, Powell JF, Breakefield XO, Sims KB, et al Isolation and characterization of a candidate gene for Norrie disease. Nat Genet. 1992;1(3):204–8. . 1303236 10.1038/ng0692-204

[25] Dickinson JL, Sale MM, Passmore A, FitzGerald LM, Wheatley CM, Burdon KP, et al Mutations in the NDP gene: contribution to Norrie disease, familial exudative vitreoretinopathy and retinopathy of prematurity. Clin Experiment Ophthalmol. 2006;34(7):682–8. . 16970763 10.1111/j.1442-9071.2006.01314.x

[26] Meindl A, Berger W, Meitinger T, van de Pol D, Achatz H, Dorner C, et al Norrie disease is caused by mutations in an extracellular protein resembling C-terminal globular domain of mucins. Nat Genet. 1992;2(2):139–43. 1303264 10.1038/ng1092-139

[27] Collin RW, Nikopoulos K, Dona M, Gilissen C, Hoischen A, Boonstra FN, et al ZNF408 is mutated in familial exudative vitreoretinopathy and is crucial for the development of zebrafish retinal vasculature. Proceedings of the National Academy of Sciences of the United States of America. 2013;110(24):9856–61. Epub 2013/05/30. 10.1073/pnas.1220864110 23716654 PMC3683717

[28] Hu H, Xiao X, Li S, Jia X, Guo X, Zhang Q. KIF11 mutations are a common cause of autosomal dominant familial exudative vitreoretinopathy. Br J Ophthalmol. 2016;100(2):278–83. 10.1136/bjophthalmol-2015-306878 . 26472404

[29] Robitaille JM, Gillett RM, LeBlanc MA, Gaston D, Nightingale M, Mackley MP, et al Phenotypic overlap between familial exudative vitreoretinopathy and microcephaly, lymphedema, and chorioretinal dysplasia caused by KIF11 mutations. JAMA Ophthalmol. 2014;132(12):1393–9. 10.1001/jamaophthalmol.2014.2814 . 25124931

[30] Shastry BS, Pendergast SD, Hartzer MK, Liu X, Trese MT. Identification of missense mutations in the Norrie disease gene associated with advanced retinopathy of prematurity. Arch Ophthalmol. 1997;115(5):651–5. 9152134 10.1001/archopht.1997.01100150653015

[31] Hiraoka M, Berinstein DM, Trese MT, Shastry BS. Insertion and deletion mutations in the dinucleotide repeat region of the Norrie disease gene in patients with advanced retinopathy of prematurity. J Hum Genet. 2001;46(4):178–81. . 11322656 10.1007/s100380170085

[32] Haider MZ, Devarajan LV, Al-Essa M, Kumar H. A C597—>A polymorphism in the Norrie disease gene is associated with advanced retinopathy of prematurity in premature Kuwaiti infants. Journal of biomedical science. 2002;9(4):365–70. . 12145535 10.1007/BF02256593

[33] Talks SJ, Ebenezer N, Hykin P, Adams G, Yang F, Schulenberg E, et al De novo mutations in the 5' regulatory region of the Norrie disease gene in retinopathy of prematurity. J Med Genet. 2001;38(12):E46 . 11748312 10.1136/jmg.38.12.e46 PMC1734786

[34] Hutcheson KA, Paluru PC, Bernstein SL, Koh J, Rappaport EF, Leach RA, et al Norrie disease gene sequence variants in an ethnically diverse population with retinopathy of prematurity. Mol Vis. 2005;11:501–8. 16052165

[35] Hiraoka M, Takahashi H, Orimo H, Hiraoka M, Ogata T, Azuma N. Genetic screening of Wnt signaling factors in advanced retinopathy of prematurity. Mol Vis. 2010;16:2572–7. . 21151595 PMC3000231

[36] Ells A, Guernsey DL, Wallace K, Zheng B, Vincer M, Allen A, et al Severe retinopathy of prematurity associated with FZD4 mutations. Ophthalmic genetics. 2010;31(1):37–43. Epub 2010/02/10. 10.3109/13816810903479834 . 20141357

[37] MacDonald ML, Goldberg YP, Macfarlane J, Samuels ME, Trese MT, Shastry BS. Genetic variants of frizzled-4 gene in familial exudative vitreoretinopathy and advanced retinopathy of prematurity. Clin Genet. 2005;67(4):363–6. . 15733276 10.1111/j.1399-0004.2005.00408.x

[38] Kondo H, Kusaka S, Yoshinaga A, Uchio E, Tawara A, Tahira T. Genetic variants of FZD4 and LRP5 genes in patients with advanced retinopathy of prematurity. Molecular vision. 2013;19:476–85. Epub 2013/02/27. 23441120 PMC3580992

[39] Ye X, Wang Y, Cahill H, Yu M, Badea TC, Smallwood PM, et al Norrin, frizzled-4, and Lrp5 signaling in endothelial cells controls a genetic program for retinal vascularization. Cell. 2009;139(2):285–98. Epub 2009/10/20. 10.1016/j.cell.2009.07.047 19837032 PMC2779707

[40] Xu Q, Wang Y, Dabdoub A, Smallwood PM, Williams J, Woods C, et al Vascular development in the retina and inner ear: control by Norrin and Frizzled-4, a high-affinity ligand-receptor pair. Cell. 2004;116(6):883–95. . 15035989 10.1016/s0092-8674(04)00216-8

[41] Wang Y, Huso D, Cahill H, Ryugo D, Nathans J. Progressive cerebellar, auditory, and esophageal dysfunction caused by targeted disruption of the frizzled-4 gene. J Neurosci. 2001;21(13):4761–71. . 11425903 10.1523/JNEUROSCI.21-13-04761.2001 PMC6762346

[42] Stahl A, Connor KM, Sapieha P, Willett KL, Krah NM, Dennison RJ, et al Computer-aided quantification of retinal neovascularization. Angiogenesis. 2009;12(3):297–301. Epub 2009/09/17. 10.1007/s10456-009-9155-3 19757106 PMC4005267

[43] Smith LE, Wesolowski E, McLellan A, Kostyk SK, D'Amato R, Sullivan R, et al Oxygen-induced retinopathy in the mouse. Invest Ophthalmol Vis Sci. 1994;35(1):101–11. . 7507904

[44] Chen J, Michan S, Juan AM, Hurst CG, Hatton CJ, Pei DT, et al Neuronal sirtuin1 mediates retinal vascular regeneration in oxygen-induced ischemic retinopathy. Angiogenesis. 2013;16(4):985–92. 10.1007/s10456-013-9374-5 23912262 PMC4006695

[45] Stahl A, Connor KM, Sapieha P, Chen J, Dennison RJ, Krah NM, et al The mouse retina as an angiogenesis model. Invest Ophthalmol Vis Sci. 2010;51(6):2813–26. 10.1167/iovs.10-5176 20484600 PMC2891451

[46] Holmes JM, Duffner LA. The effect of postnatal growth retardation on abnormal neovascularization in the oxygen exposed neonatal rat. Curr Eye Res. 1996;15(4):403–9. Epub 1996/04/01. . 8670740 10.3109/02713689608995831

[47] Vanhaesebrouck S, Daniels H, Moons L, Vanhole C, Carmeliet P, De Zegher F. Oxygen-induced retinopathy in mice: amplification by neonatal IGF-I deficit and attenuation by IGF-I administration. Pediatr Res. 2009;65(3):307–10. Epub 2008/12/19. 10.1203/PDR.0b013e3181973dc8 . 19092722

[48] Kondo H, Kusaka S, Yoshinaga A, Uchio E, Tawara A, Tahira T. Genetic variants of FZD4 and LRP5 genes in patients with advanced retinopathy of prematurity. Mol Vis. 2013;19:476–85. Epub 2013/02/27. 23441120 PMC3580992

[49] Ohlmann A, Seitz R, Braunger B, Seitz D, Bosl MR, Tamm ER. Norrin promotes vascular regrowth after oxygen-induced retinal vessel loss and suppresses retinopathy in mice. J Neurosci. 2010;30(1):183–93. Epub 2010/01/08. 10.1523/JNEUROSCI.3210-09.2010 . 20053900 PMC6632540

[50] Tokunaga CC, Chen YH, Dailey W, Cheng M, Drenser KA. Retinal vascular rescue of oxygen-induced retinopathy in mice by norrin. Invest Ophthalmol Vis Sci. 2013;54(1):222–9. Epub 2012/11/29. 10.1167/iovs.12-10127 . 23188723

[51] Early Treatment for Retinopathy of Prematurity Cooperative G, Good WV, Hardy RJ, Dobson V, Palmer EA, Phelps DL, et al Final visual acuity results in the early treatment for retinopathy of prematurity study. Archives of ophthalmology. 2010;128(6):663–71. Epub 2010/04/14. 10.1001/archophthalmol.2010.72 20385926 PMC4162423

[52] Siatkowski RM, Good WV, Summers CG, Quinn GE, Tung B. Clinical characteristics of children with severe visual impairment but favorable retinal structural outcomes from the Early Treatment for Retinopathy of Prematurity (ETROP) study. J AAPOS. 2013;17(2):129–34. Epub 2013/03/26. 10.1016/j.jaapos.2012.10.022 . 23522948 PMC4381920

[53] Quinn GE, Dobson V, Hardy RJ, Tung B, Palmer EA, Good WV, et al Visual field extent at 6 years of age in children who had high-risk prethreshold retinopathy of prematurity. Arch Ophthalmol. 2011;129(2):127–32. Epub 2011/02/16. 10.1001/archophthalmol.2010.360 . 21320954

[54] Andersen CC, Phelps DL. Peripheral retinal ablation for threshold retinopathy of prematurity in preterm infants. The Cochrane database of systematic reviews. 2000;(2):CD001693 Epub 2000/05/05. 10.1002/14651858.CD001693 . 10796444 PMC8406950

[55] Tahija SG, Hersetyati R, Lam GC, Kusaka S, McMenamin PG. Fluorescein angiographic observations of peripheral retinal vessel growth in infants after intravitreal injection of bevacizumab as sole therapy for zone I and posterior zone II retinopathy of prematurity. Br J Ophthalmol. 2014;98(4):507–12. Epub 2014/01/10. 10.1136/bjophthalmol-2013-304109 24403566 PMC3963534

[56] Lepore D, Quinn GE, Molle F, Baldascino A, Orazi L, Sammartino M, et al Intravitreal bevacizumab versus laser treatment in type 1 retinopathy of prematurity: report on fluorescein angiographic findings. Ophthalmology. 2014;121(11):2212–9. Epub 2014/07/09. 10.1016/j.ophtha.2014.05.015 . 25001158

[57] Chen W, Binenbaum G, Karp K, Baumritter A, Pearson DJ, Maguire AM, et al Late recurrence of retinopathy of prematurity after treatment with both intravitreal bevacizumab and laser. J AAPOS. 2014;18(4):402–4. Epub 2014/08/05. 10.1016/j.jaapos.2014.03.011 25087645 PMC4277745

[58] Henaine-Berra A, Garcia-Aguirre G, Quiroz-Mercado H, Martinez-Castellanos MA. Retinal fluorescein angiographic changes following intravitreal anti-VEGF therapy. J AAPOS. 2014;18(2):120–3. Epub 2014/04/05. 10.1016/j.jaapos.2013.12.009 . 24698606

[59] Mehta S, Hubbard GB 3rd. Delayed recurrent neovascularization and persistent avascular retina following intravitreal bevacizumab for retinopathy of prematurity. Retinal cases & brief reports. 2013;7(3):206–9. Epub 2013/07/01. 10.1097/ICB.0b013e318285238e . 25391107

[60] Ittiara S, Blair MP, Shapiro MJ, Lichtenstein SJ. Exudative retinopathy and detachment: a late reactivation of retinopathy of prematurity after intravitreal bevacizumab. J AAPOS. 2013;17(3):323–5. Epub 2013/04/24. 10.1016/j.jaapos.2013.01.004 . 23607977

[61] Kong L, Bhatt AR, Demny AB, Coats DK, Li A, Rahman EZ, et al Pharmacokinetics of Bevacizumab and Its Effects on Serum VEGF and IGF-1 in Infants With Retinopathy of Prematurity. Invest Ophthalmol Vis Sci. 2015;56(2):956–61. Epub 2015/01/24. 10.1167/iovs.14-15842 . 25613938

[62] Sato T, Wada K, Arahori H, Kuno N, Imoto K, Iwahashi-Shima C, et al Serum concentrations of bevacizumab (avastin) and vascular endothelial growth factor in infants with retinopathy of prematurity. Am J Ophthalmol. 2012;153(2):327–33 e1. Epub 2011/09/21. 10.1016/j.ajo.2011.07.005 . 21930258

[63] Spandau U, Tomic Z, Ewald U, Larsson E, Akerblom H, Holmstrom G. Time to consider a new treatment protocol for aggressive posterior retinopathy of prematurity? Acta Ophthalmol. 2013;91(2):170–5. Epub 2012/01/25. 10.1111/j.1755-3768.2011.02351.x . 22268644

[64] Avery RL. Bevacizumab (Avastin) for retinopathy of prematurity: wrong dose, wrong drug, or both? J AAPOS. 2012;16(1):2–4. Epub 2012/01/13. 10.1016/j.jaapos.2011.11.002 . 22237669

[65] Darlow BA, Ells AL, Gilbert CE, Gole GA, Quinn GE. Are we there yet? Bevacizumab therapy for retinopathy of prematurity. Archives of disease in childhood Fetal and neonatal edition. 2013;98(2):F170–4. Epub 2012/01/03. 10.1136/archdischild-2011-301148 . 22209748

