# Supplementary material for: Fzd4 Haploinsufficiency Delays Retinal Revascularization in the Mouse Model of Oxygen Induced Retinopathy
Source: PLoS One. 2016 Aug 4;11(8):e0158320. doi: 10.1371/journal.pone.0158320 (PMC4973993; doi:10.1371/journal.pone.0158320)
Supplement: S1 Table — (PDF) [file pone.0158320.s001.pdf]

Age	P12	P12	P12	P12	P17	P17	P17	P17	P25	P25	P25	P25
Treatment	Normoxic	Normoxic	OIR	OIR	Normoxic	Normoxic	OIR	OIR	Normoxic	Normoxic	OIR	OIR
Genotype	$FZD4^{+/+}$	$FZD4^{+/-}$	$FZD4^{+/+}$	$FZD4^{+/-}$	$FZD4^{+/+}$	$FZD4^{+/-}$	$FZD4^{+/+}$	$FZD4^{+/-}$	$FZD4^{+/+}$	$FZD4^{+/-}$	$FZD4^{+/+}$	$FZD4^{+/-}$
Avg. Weight (g)	5.68	5.58	6.8	6.12	7.97	9.46	8.23	8.3	11.8	11.45	13.96	12.2
St. Error	0.38	0.32	0.91	0.87	2.1	2.2	1.8	2.1	0.6	0.4	0.98	2.8
n	7	7	6	7	6	8	6	7	7	11	8	7

Table S1
